# Physical Exercise following bariatric surgery in women with Morbid obesity: Study protocol clinical trial (SPIRIT compliant)

**DOI:** 10.1097/MD.0000000000019427

**Published:** 2020-03-20

**Authors:** Alberto Soriano-Maldonado, Sonia Martínez-Forte, Manuel Ferrer-Márquez, Elena Martínez-Rosales, Alba Hernández-Martínez, Alejandro Carretero-Ruiz, Emilio Villa-González, Yaira Barranco-Ruiz, Manuel A. Rodríguez-Pérez, María José Torrente-Sánchez, Lorena Carmona-Rodríguez, Pablo Soriano-Maldonado, José A. Vargas-Hitos, Antonio J. Casimiro-Andújar, Enrique G. Artero, Ana M. Fernández-Alonso

**Affiliations:** a Department of Education, Faculty of Education Sciences; and SPORT Research Group (CTS-1024), CERNEP Research Center, University of Almería; b Obstetrics and Gynecology Unit, Torrecárdenas University Hospital, Almería; c Bariatric Surgery Department, Torrecárdenas University Hospital, Almería; d Obesidad Almería, Hospital Mediterráneo, Almería; e Department of Physical and Sports Education, PROFITH “PROmoting FITness and Health through Physical Activity” Research Group, Sport and Health University Research Institute (iMUDS), Faculty of Education and Sport Sciences, University of Granada, Melilla; f Department of Macromolecular Structures, Proteomics Unit, Centro Nacional de Biotecnología (CNB/CSIC); g Structural Biology Program, Spanish National Cancer Research Centre (CNIO), Madrid; h Systemic Autoimmune Diseases Unit, Department of Internal Medicine, “Virgen de las Nieves” University Hospital, Granada, Spain.

**Keywords:** arterial stiffness, bariatric surgery, cardiorespiratory fitness, exercise, fertility, inflammation, morbid obesity

## Abstract

**Background:**

Severe and morbid obesity are increasing globally, particularly in women. As BMI increases, the likelihood of anovulation is higher. The primary aim of the EMOVAR clinical trial is to examine, over the short (16 weeks) and medium (12 months) term, the effects of a supervised physical exercise program (focused primarily on aerobic and resistance training) on ovarian function in women with severe/morbid obesity who have undergone bariatric surgery. Secondary objectives are to examine the effects of the intervention on chronic inflammation, insulin resistance, arterial stiffness, physical fitness, and health-related quality of life.

**Methods:**

This is a randomized controlled trial in which ∼40 female bariatric surgery patients, aged between 18 and 45 years old, will be included. Participants assigned to the experimental group will perform a total of 48 sessions of supervised concurrent (strength and aerobic) training (3 sessions/week, 60 min/session) spread over 16 weeks. Patients assigned to the control group will receive lifestyle recommendations. Outcomes will be assessed at baseline, week 16 (i.e., after the exercise intervention) and 12 months after surgery. The primary outcome is ovarian function using the Sex-Hormone Binding Globuline, measured in serum. Secondary outcomes are serum levels of anti-mullerian hormone, TSH, T4, FSH, LH, estradiol, prolactine, and free androgen index, as well as oocyte count, the diameters of both ovaries, endometrial thickness, and uterine arterial pulsatility index (obtained from a transvaginal ultrasound), the duration of menstrual bleeding and menstrual cycle duration (obtained by personal interview) and hirsutism (Ferriman Gallwey Scale). Other secondary outcomes include serum markers of chronic inflammation and insulin resistance (i.e., C-reactive protein, interleukin 6, tumor necrosis factor-alpha, leptin, glomerular sedimentation rate, glucose, insulin and the HOMA-IR), arterial stiffness, systolic, diastolic and mean blood pressure, body composition, and total weight loss. Physical fitness (including cardiorespiratory fitness, muscular strength, and flexibility), health-related quality of life (SF-36 v2) and sexual function (Female Sexual Function Index) will also be measured.

**Discussion:**

This study will provide, for the first time, relevant information on the effects of exercise training on ovarian function and underlying mechanisms in severe/morbid obese women following bariatric surgery.

**Trial registration number:**

ISRCTN registry (ISRCTN27697878).

## 1 Introduction

The prevalence of severe/morbid obesity (body mass index; BMI ≥35 kg/m^2^) has increased globally in recent decades,^[1–4]^ being close to 7.5% in the United States.^[5]^ In Spain, figures show that the prevalence of morbid obesity (BMI ≥40 kg/m^2^) increased by more than 200% in the last 3 decades,^[3]^ being 1.39% in women (i.e., >300,000 cases) in 2016,^[6]^ and with specific areas of Spain presenting a prevalence of severe and morbid obesity (combined) of up to 10%.^[7]^ Cardiometabolic risk factors are significantly altered in patients with severe/morbid obesity, even compared with patients with type I obesity (BMI ≥30 and <35), both in men and women,^[7]^ which partially justifies the higher incidence of morbidity (e.g., hypertension, type 2 diabetes, metabolic syndrome, etc), cardiovascular events and cardiovascular^[8]^ and all-cause^[9,10]^ mortality (i.e., up to 30% higher compared to normal weight individuals^[10]^) observed in this population.

According to the National Statistical Institute (INE), the number of births in 2017 decreased by 4.5% compared to 2016,^[11]^ and by more than 20% compared to 2008. Severe/morbid obesity produces significant gynaecological problems including ovulatory problems,^[12]^ which contribute to the decline in birth rates. Increased adiposity is associated with irregularities in menstruation, anovulation, hirsutism, and polycystic ovary syndrome, among others,^[12,13]^ which results in significant problems with ovulating, subfertility and infertility^[13]^; this not only affects women's gynaecological health, but also their quality of life and potentially their relationship with a partner. The mechanisms that link obesity to ovarian function are not yet known in depth, although it appears that there is an alteration in the hypothalamus–pituitary–ovarian axis.^[14]^ Excess adiposity increases the aromatization of androgens to estrogen, decreasing the hepatic synthesis of sex hormone-binding globulin (SHBG).^[13,15]^ This increases levels of estradiol and circulating testosterone (raising the free androgenic index), and thus increases the risk of menstruation irregularities. Reduced pulsatility of luteinising hormone (LH) has been observed, which could reduce oocyte recruitment and quality and alter endometrial decidualization, which in turn could affect the function of the corpus luteum during the luteal phase^[16]^ and alter oocyte maturation.^[17]^ In addition, obesity appears to promote the accumulation of lipids in the oocyte, which activates the stress pathway in the endoplasmic reticulum, inducing mitochondrial dysfunction and increased apoptosis of the developing ovarian follicles.^[18]^

Many of the ovulatory problems observed in women with severe/morbid obesity may be mediated by a chronic pro-inflammatory status (excessive accumulation of pro-inflammatory cytokines)^[19]^ that favor, for instance, the incidence of polycystic ovary syndrome^[20,21]^ making ovulation difficult. In obese women, Jungheim et al observed noticeable alterations of inflammatory markers, such as C-reactive protein, leptin, or tumor necrosis factor alpha, in the follicular fluid.^[16]^ Furthermore, obesity is associated with vascular alterations and elevated arterial stiffness,^[22–25]^ which is associated with significant changes in uterine artery flow^[25]^ and could be related to ovulatory problems.

For many women with severe/morbid obesity, bariatric surgery^[26,27]^ is the first step towards a change in their lifestyle and quality of life, as it significantly reduces excess weight^[28]^ and promotes the remission of metabolic syndrome, hypertension or type 2 diabetes,^[29]^ which in turn significantly decreases the incidence of morbidity–mortality.^[30,31]^ Bariatric surgery seems to improve a woman's ovarian function by reducing the free androgen index, partially improving the luteal function,^[32]^ regulating the menstrual cycle (by increasing follicle-stimulating hormone [FSH] and LH, and decreasing testosterone and Dehydroepiandrosterone sulfate [DHEA-S]), improving conception rates and reducing the incidence of early abortions.^[33]^ However, other authors indicate that by increasing the rate of maternal malabsorption (depending on the surgical technique) and decreasing the ovarian reserve by decreasing levels of Anti-Müllerian hormone,^[34,35]^ fertility could continue to be compromised following bariatric surgery. Moreover, the success of bariatric surgery and the evolution of the patient in the short, (and above all) medium and long term is strongly determined by their post-operative lifestyle.^[36]^ About 50% of patients regain weight in the 24 months after surgery^[37]^ and may again have complications associated with severe/morbid obesity.^[38,39]^ It is therefore of great clinical and public health importance to study the effectiveness of complementary interventions aimed at improving the lifestyle of the obese patient from the first year of bariatric surgery in order to understand the effects of these changes in ovarian function and reproductive capacity, compared to the usual post-operative treatment.

Physical exercise following bariatric surgery has been shown to significantly improve cardiorespiratory fitness^[40]^ and muscular strength,^[41,42]^ to regulate autonomic nervous system dysfunction^[40,43]^ and to improve mitochondrial respiration at the muscular level^[44]^ as well as glucose metabolism.^[44,45]^ Our research group observed that being physically active is associated with a more favorable cardiometabolic profile in women with severe/morbid obesity,^[7]^ and has recently suggested^[46]^ that physical exercise can be a powerful predictor of the improvements observed in pain and physical function following bariatric surgery.^[47]^ Supervised physical exercise could also have positive effects on ovarian function following bariatric surgery. In women of reproductive age, exercise has shown to be effective in improving ovulatory cycles, ovulation and fertility, decreasing testosterone and the free androgen index, and increasing SHBG in the absence of obesity.^[48]^ In obese women, exercise similarly appears to increase the number of ovulatory cycles and improve fertility, despite absence of weight loss.^[49]^ For obese women with polycystic ovary syndrome, Palomba et al^[50]^ demonstrated that exercise reduces insulin-resistance in a more lasting way than diet (reducing the amount of adipose tissue and improving glucose metabolism in skeletal muscle), restoring ovulation in 65% of the participants.^[50]^ In this line, Coen et al,^[44,45]^ in a sample of 128 participants (88% women) with severe/morbid obesity undergoing bariatric surgery, demonstrated that partially supervised exercise increases insulin sensitivity with respect to a group receiving usual care, although there were no changes in weight loss. In conjunction, these results indicate that the effects of exercise on insulin resistance are, at least partially, independent of weight loss, and suggest that exercise could reduce the androgenic profile (free testosterone, dihydroepiandrostendione, and the free androgen index) and increase SHBG production, improving or even restoring ovulation.^[51]^ Hakimi et al^[52]^ systematically reviewed the effects of exercise on ovulation and concluded that 30 to 60 min/day of vigorous exercise is associated with a lower risk of anovulation, and that exercise can be used as a treatment for anovulation in overweight and obese women.

Other proposed mechanisms implicated in the relationship between obesity and reproductive problems, such as chronic inflammation^[53]^ or arterial stiffness,^[54]^ could also be significantly improved through exercise in this population. Recently, Stolberg et al^[55]^ found no improvement in inflammation following a training program initiated 6 months after bariatric surgery (70% women), although program adherence was clearly insufficient (i.e., only 59.4% of the participants attended 50% or more sessions) and the insufficient details about the intervention compromises replicability and the quality of the study.^[56]^ Therefore, the extent to which exercise, started immediately after surgery, can improve inflammation and arterial distensibility, and whether changes in these parameters can mediate possible improvements in women's ovarian function remain to be investigated.

For all of the above, studying the effectiveness of a supervised concurrent (strength and aerobic) exercise programme on the ovarian function of women undergoing bariatric surgery, both in the short (16 weeks) and medium (12 months) term, is of great clinical and social interest, especially considering the significant ovulatory problems that obesity confers. We postulate that a supervised evidence-based exercise program following the *Consensus on Exercise Reporting Template* (CERT),^[57,58]^ significantly improves ovarian function following bariatric surgery.

Thus, the main aim of the EMOVAR clinical trial is to examine, over the short (16 weeks) and medium (12 months) term, the effects of a supervised physical exercise program (focused primarily on aerobic and resistance training) on ovarian function in women with severe/morbid obesity who have undergone bariatric surgery, in comparison with usual care following bariatric surgery. Secondary aims are to examine the effects of the intervention on chronic inflammation and insulin resistance, arterial stiffness, physical fitness and health-related quality of life. We will also assess whether changes in inflammatory parameters, insulin resistance or arterial stiffness, induced by exercise, are associated with changes in ovarian function. Finally, the extent to which changes in body weight and composition, or changes in physical fitness (i.e., aerobic capacity and muscular strength), induced by the training program, are associated with changes in ovarian function will also be investigated.

## 2 Material and Methods

### 2.1 Study design and protocol registration

The EMOVAR study is a parallel-group randomized controlled trial (RCT) registered at the ISRCTN registry (ISRCTN27697878) on October 4, 2019, before the enrolment of participants begun (i.e., on October 15, 2019). The study protocol (version 1.0) was reviewed and approved by the Ethics Committee of the Torrecárdenas University Hospital (code RTI2018-093302-A-I00; 7/2019) on January 30, 2019.

### 2.2 Recruitment and eligibility criteria

#### 2.2.1 Recruitment

Participants will be recruited through the Bariatric Surgery Unit at Torrecárdenas University Hospital (the main referral hospital in the city of Almería, southern Spain) and Hospital Mediterráneo, consecutively one at a time, during the pre-surgery appointments. In both hospitals, the equipment, the criteria to indicate bariatric surgery, as well as the techniques performed, are identical. These two hospitals cover ∼90% to 95% of the bariatric surgeries performed in the entire province of Almería. Under normal conditions, ∼80 to 90 patients (∼70% women) attend the clinics (including both centres) annually, of which around 70 to 75 patients meet the bariatric surgery criteria and are eventually intervened.

#### 2.2.2 Eligibility criteria

The inclusion and exclusion criteria are detailed in Table 1.

**Table 1:**
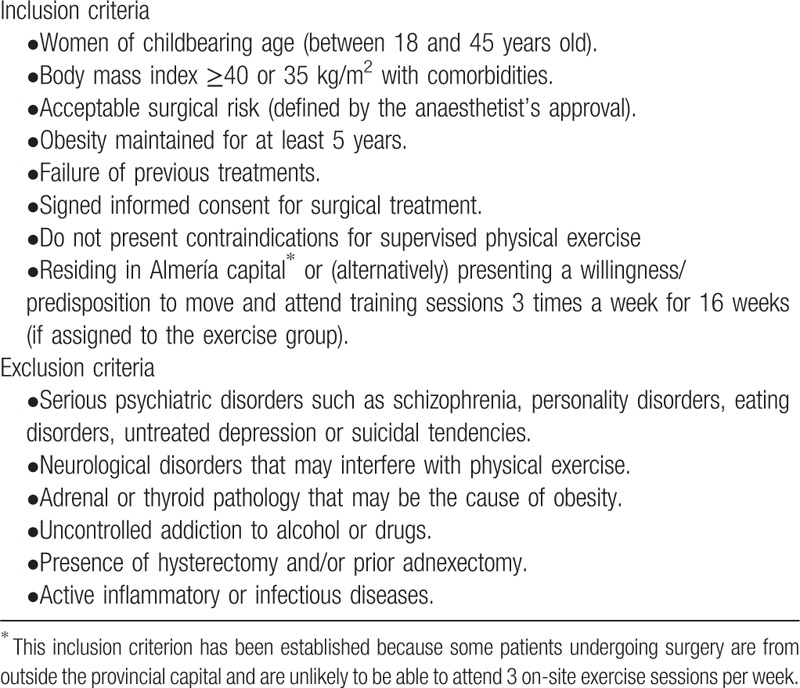
Inclusion and exclusion criteria for selecting participants.

### 2.3 Sample size

**Figure 1:**
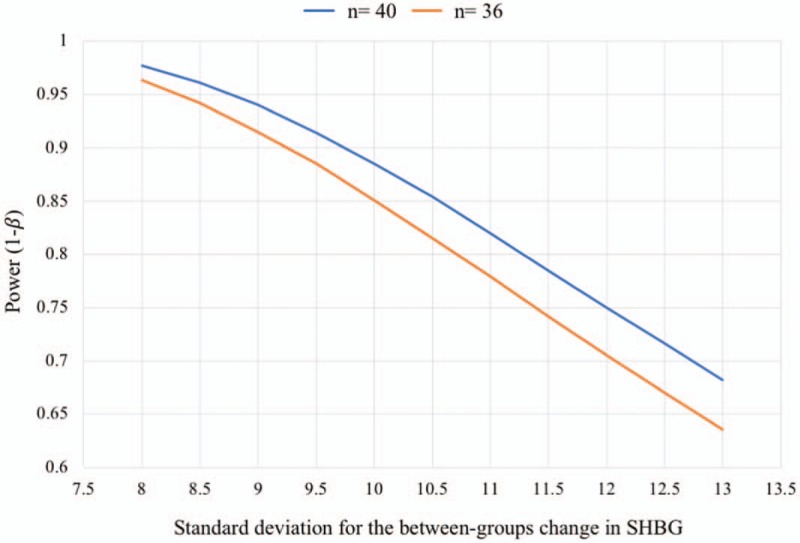
Study power curve to detect a 10 nmol/L increase in the primary outcome (SHBG) for different standard deviations of the between-group difference in the change from baseline.

The number of patients needed was estimated based on the primary outcome—SHBG. At the time this protocol was conceived, there were no previous studies on the effects of physical exercise on ovarian function in women with severe/morbid obesity or after undergoing bariatric surgery. Therefore, the sample size was estimated using prior research on exercise effects in obese women,^[59]^ based on an expected increase in SHBG of at least 10 nmol/L in the EG compared to the CG. To detect a between-group change of 10 nmol/L (assuming a standard deviation equal to the expected effect), with a statistical power of 85% and an α error of 0.05, 36 women (n = 18 per group) will be needed. Anticipating a 10% follow-up loss, we will aim at recruiting ∼40 women. Adherence strategies will be implemented during the intervention program to minimize potential follow-up losses. If no participants are lost to follow-up, the final power of the study (with n = 40) for detecting the indicated effect would be 88.5%. In the absence of an accurate estimate for the expected standard deviation, Figure 1 represents the power curve for estimating the expected effect (the variation in the SHBG change between the EG and the CG of 10 nmol/L), considering different standard deviations and for a sample size of 36 (n ∼ 18 per group) or 40 (n ∼ 20 per group).

### 2.4 Randomization

**Figure 2:**
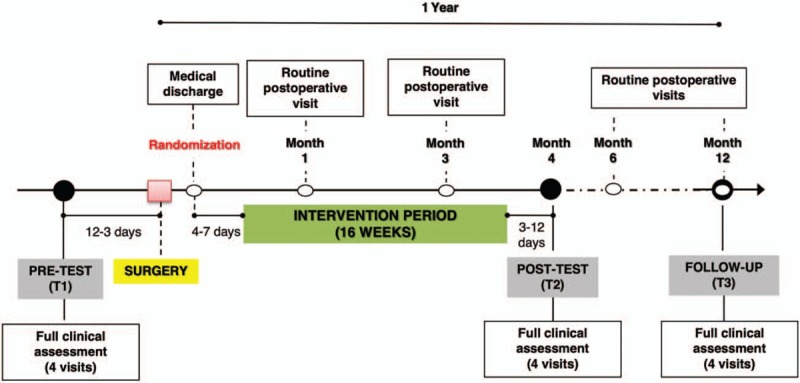
Graphical representation of the data-collection planning.

The patients will be randomly assigned either to supervised physical exercise (exercise group; EG) or to a usual care control group (CG). A simple computer-generated randomization sequence^[60]^ will be obtained, representing the allocation of each participant. Individual allocations will be held into a sealed, opaque envelope and numbered in sequential order corresponding to the order in which the participants will be randomized. Each participant will be randomized (the corresponding envelope will be opened in front of the patient) at medical discharge following bariatric surgery, provided the participant has previously met the inclusion criteria, signed the informed consent, and performed baseline assessments (pre-test; Fig. 2). All study-related information will be stored securely at the study site. All laboratory specimens, reports, data collection, process, and administrative forms will be identified by a coded ID (identification) number to maintain participants’ confidentiality. All records that contain names or other personal identifiers, such as locator forms and informed consent forms, will be stored separately from study records identified by code number and only accessible by the principal investigators.

### 2.5 Blinding

Due to the nature of the intervention (physical exercise), it is not possible to “blind” the patients, since they will inevitably know their allocation. However, the team that evaluates the primary and secondary outcomes, including the gynaecological tests, will be blinded to the patients’ allocation. The participants will receive explicit instructions not to disclose their allocation with the study staff. In addition, the data analysts will also be blinded to the patients’ allocation.

### 2.6 Data collection

#### 2.6.1 Assessment calendar

Figure 2 graphically represents the data-collection planning that each enrolled participant will complete throughout the study. All participants will complete the whole battery of assessments at three time points (i.e., at baseline or pre-test [T1], within ∼12 days before the surgery; week 16 or post-test [T2], within a margin of 3 to 12 days following the intervention completion; and 12 months or follow-up [T3], within a 15 day margin).

Each of the 3 full clinical assessments (pre-test, post-test, and 12-month follow-up) consists of 4 visits (Table 2). In addition, participants in both groups will continue their protocol of regular visits to the surgical and nutrition clinics during the study (months 1, 3, 6, and 12 after surgery), in which participants from both groups (EG and CG) will receive lifestyle recommendations including internationally accepted physical activity and nutrition recommendations.^[27]^

**Table 2:**
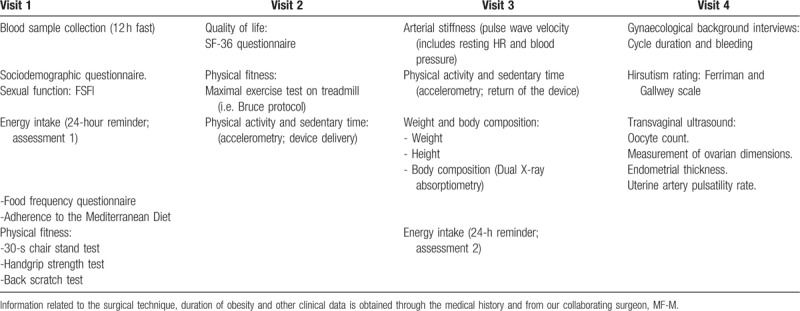
Outline of the planned evaluations during each of the 3 full study assessments (pre-test, post-test, and follow-up at 12 months).

#### 2.6.2 Data collection related to the primary study aim (ovarian function)

All the variables measured in serum will come from a blood sample (day 1 of assessment) taken in the morning (9:00) after a minimum of 12 h fasting. 20 mL of blood will be taken, which will then be processed, frozen and stored in a standardized way in the Public Health System of Andalusia Biobank (https://goo.gl/SbGFyd) for further analysis, thus guaranteeing the standardization and quality of the biological sampling process.

##### 2.6.2.1 Primary outcome

The primary study outcome is the SHBG (nmol/L) since it is a fundamental hormone for ovarian function and ovulation, is inversely related to obesity and is likely to increase with weight loss and physical exercise. It will be measured in serum and analyzed by immunoassay using the Beckman Coulter kit (ref. A48617), with a maximum value of 200 nmol/L and an inaccuracy of <7%.

##### 2.6.2.2 Secondary outcomes related to ovarian function

###### 2.6.2.2.1 Serum markers

Anti-Müllerian hormone (AMH, ng/mL; kit's ref. B13127), follicle-stimulating hormone (FSH, mIU/mL; kit's ref. 33520), thyroxine (T4, ng/dL; kit's ref. 33880), thyrotropin (TSH, μIU/mL; kit's ref. B63284), LH (mUI/mL; kit's ref. 33510), estradiol (E2, pmol/L; kit's ref. B84493), prolactin (PRL, ng/mL; kit's ref. 33530) and total testosterone (T, nmol/L; kit's ref. 33560) will be measured by chemiluminiscence immunoassay (Beckman Coulter) according to manufacturer's protocols. Finally, free androgen index will be calculated as the total testosterone/SHBG ratio.

###### 2.6.2.2.2 Markers obtained from a transvaginal ultrasound using Toshiba Xario ultrasound equipment (Toshiba Medical Systems Corporation, Japan) equipped with a 7 MHz curved endovaginal transducer

- Oocyte count. The total number of follicles of between 2 and 8 mm in size displayed on both ovaries will be recorded.
- Ovarian volume: the longitudinal, oblique, and transverse axes are measured graphically in millimetres. The ovarian volume of each ovary is calculated with the following formula: volume = D1 × D2 × D3 × 0.52.^[61]^ Where D represents the longitudinal, oblique and transverse axes, respectively.
- Endometrial thickness: the thickness of the endometrium (measured in millimetres) displayed ultrasonically in a longitudinal section of the uterus.
- Uterine arterial pulsatility index (left, right, and middle), measured using ultrasound. The 7 MHz endovaginal transducer should be located paramedial to the uterine cervix at the level of the inner cervical opening. The vessel should be identified with colour Doppler using high speed scales (between 30 and 50 cm/s) to allow selective identification. The insertion angle for the measurements must be less than 45°. Three or more waves of similar characteristics should be obtained for the measurement, with adequate magnification, occupying at least three quarters of the screen. The size of the Doppler sample should be equivalent to the artery's diameter and placed in the centre of the vessel. The pulsatility index (PI) is then calculated.

###### 2.6.2.2.3 Other outcomes relevant to the primary aim (ovarian function)

- The duration of menstrual bleeding (days) and menstrual cycles (days) will be obtained through interview.
- Hirsutism. This will be measured using the Ferriman Gallwey Scale.^[62]^ The score will be: less than or equal to 8—normal; from 8 to 11—mild hirsutism; from 12 to 19—moderate hirsutism; and from 20 and above—severe hirsutism.

#### 2.6.3 Data collection related to secondary aims

##### 2.6.3.1 Markers of chronic inflammation and insulin resistance obtained from serum

- High-sensitivity C-reactive protein (hs-CRP) levels (mg/L), as a relevant indicator of systemic inflammation which is highly susceptible to decrease through physical exercise.^[63]^ This is performed by immunoturbidimetric analysis using the Beckman Coulter kit, with a detection limit of 80 mg/L and a coefficient of variation <1 mg/L.
- Interleukin 6 (IL-6; pg/mL), tumor necrosis factor alpha (TNFα; pg/mL) and leptin (pg/mL) will be measured by immunoassay using specific kits according to manufacturer's protocols: Human IL-6 ELISA Kit High Sensitivity (abcam; ab46042), TNF alpha Human ELISA Kit (Thermo Fisher Scientific; KHC3011) and Leptin Human ELISA Kit (Thermo Fisher Scientific; KAC2281), respectively.
- Glomerular sedimentation rate (GSR, mm/h). This will be measured using the Menarini Diagnostics reagent.
- Glucose and insulin. Glucose and insulin will be measured by immunoassay using Cobas kit according to manufacturer's protocols. The HOMA (homeostasis model assessment of insulin resistance) will be calculated using the formula ([insulin, mIU/L] × [glucose, mg/dL])/405.^[64]^

##### 2.6.3.2 Arterial stiffness and systolic, diastolic, and mean arterial blood pressure

Arterial stiffness will be measured through pulse wave velocity (PWV), the elevation of which is an early marker of arteriosclerosis.^[65]^ The Mobil-O-Graph 24 h pulse wave analysis monitor (IEM GmbH, Stolberg, Germany) will be used, the operation of which is based on the oscillometry recorded by a blood pressure sleeve placed on the brachial artery.^[66–68]^ This device has been shown to be reliable in measuring the PWV in different populations, measuring (in addition to arterial stiffness) the brachial and central blood pressure [both systolic (SBP) and diastolic (DBP)], as well as the resting heart rate in one single measurement.^[67]^ All these parameters will be measured in the same procedure and using the same device, in triplicate, in a seated position, after 5 min of rest, in a room that has a comfortable temperature, following international recommendations.^[65,69]^ Mean blood pressure (MAP) will be calculated using the formula MAP 1/3(SAP-DAP) + DAP.

##### 2.6.3.3 Body composition and total weight loss

Body composition will be assessed by dual-energy X-ray absorptiometry (DEXA; DMS Imaging, STRATOS dR). The percentage of total and segmental body fat, fat-free mass and muscle mass will be measured. Weight (InBody 270) and height (Seca 213) will be measured. Total weight loss (in %) will be calculated as (pre-operative weight—follow-up weight/pre-operative weight) × 100].^[70]^ While in non-bariatric obese adult population, a total weight loss of 10% is usually the target ^[71]^, in the bariatric surgery population, a weight loss <20% at 12 months after the surgery is considered insufficient or suboptimal.^[70]^

##### 2.6.3.4 Physical fitness

Cardiorespiratory fitness will be evaluated with the Bruce protocol^[72]^ on a treadmill, one of the most widely used tests worldwide to assess aerobic capacity.^[73]^ The lower limb muscular strength will be evaluated using the 30 s chair stand test,^[74]^ while the upper limb muscular strength will be evaluated using handgrip dynamometry.^[75]^ Finally, the back-scratch test will be performed to evaluate the range of motion of the shoulder and the shoulder girdle.^[74]^

##### 2.6.3.5 Health-related quality of life

Health-related quality of life will be evaluated with the Spanish version of the 36-item Short Form Health Survey (SF-36 v2).^[76]^ The total score of each of the 8 subscales (and the physical and mental composite scores) ranges from 0 to 100, with high values representing a higher quality of life.

##### 2.6.3.6 Sexual function

Sexual function will be evaluated using the Spanish version^[77]^ of the 9-item Female Sexual Function Index (FSFI),^[78]^ question questionnaire grouped into six domains: desire, excitement, lubrication, orgasm, satisfaction, and pain. The score of each item goes from 0–5 or 1–5, with low values representing a female sexual dysfunction.

#### 2.6.4 Control variables and other variables to be recorded

Physical activity (PA) and sedentary time will be measured by accelerometry (ActiGraph GT3x+; ActiLife software version 6.11.7).^[79,80]^ In each of the 3 full assessments, patients will wear a triaxial accelerometer on the right hip for 24 h (except for bathing or water activities) that records acceleration in all three movement axes and provides results in min/day of physical activity of different intensities and sedentary time for 7 complete days. Based on the moderate-to-vigorous physical activity, we will determine whether participants meet (or not) the American College of Sports Medicine's minimum physical activity recommendations (≥150 min/week of moderate to vigorous physical activity).

Usual intake estimates of food groups, energy and nutrients: the self-administered food frequency questionnaire (FFQ) used in the PREDIMED study^[81]^ will be employed, along with 24-h reminders on 2 non-consecutive days.

Adherence to the Mediterranean diet will be measured using the questionnaire employed in the PREDIMED study.^[82]^

Sociodemographic variables: age, marital status, educational level, employment situation, income level, among others, will all be recorded ad hoc. Relevant data from clinical history, such as personal antecedents of obesity (duration of obesity), cardiovascular disease, hypertension, obstructive sleep apnoea (OSA), type 2 diabetes, or medication use, will be recorded.

Surgical technique: the surgical techniques performed will mainly be the one anastomosis gastric bypass (OAGB), and the laparoscopic sleeve gastrectomy (SG; only when BMI ≥ 50).

### 2.7 Intervention

All the participants will continue their habitual care following bariatric surgery.

#### 2.7.1 Experimental group (supervised exercise; EG)

Patients assigned to the EG will perform a 48-session supervised physical exercise program spread over 16 weeks with a frequency of 3 sessions per week and a volume of 1 hour per session. This distribution has proven applicable in patients undergoing bariatric surgery.^[83]^ The exercise program has been comprehensively described elsewhere^[84]^ following the Consensus on Exercise Reporting Template (CERT)^[58,85]^ to maximize transparency and replicability. The exercise program will combine muscle strength and aerobic exercise in the same session (called concurrent training). Concurrent training has shown to increase weight loss and improve body composition, muscle strength, aerobic capacity and bone-mineral density to a greater extent than aerobic training and separate strength training in patients obese dieting adults.^[86]^ The exercise program will comply with international guidelines^[87,88]^ for aerobic and strength training, following criteria to progress effectively and safely.^[58]^ The training sessions will be individual and will be held in the sports facilities at the University of Almería. All sessions will be supervised^[89]^ by a Personal Trainer with a Grade in Physical Activity and Sports Sciences, and either a Master Degree in Personal Training or >2 years’ experience training obese people.

The training sessions will consist of a 5-min warm up on a treadmill, a main section of 50 min (i.e., including compensatory exercises at three levels of difficulty, followed by concurrent training), and a 5-min cool-down with dynamic and static flexibility exercises. The main section will combine a strength training block and an aerobic training block, whose exercises will be progressively complex and intense in a personalized way over 4 main phases: the familiarization phase (week 1–4), phase 1 (weeks 5–8), phase 2 (weeks 9–10) and phase 3 (weeks 11–16) (Table 3). With regard to strength training, in the familiarization phase, exercises will be carried out to learn movement patterns, strength exercises using one's own body weight and with elastic bands,^[90]^ as well as exercises to adapt to power exercises using external weight in the following phases. Starting from Phase 1, exercises will be performed with external loads and the intensity of these loads will progress approximately from 50% to 75% of 1 Repetition Maximum (1RM) quantified by the character of effort (CE), that is, based on the maximum number of repetitions that the patient can perform with each load. There will be a total of 6 main resistance exercises (i.e. squat, seated lat pull-down, bench press, seated low row, push press with dumbbells, and deadlift) focused on large muscle groups and the main movement patterns. Patients will complete 1 serie of each exercise during the first weeks of phase 1 of the program and progress to 2 and 3 series in phases 2 and 3. Rests between series will be 30–60 s and the execution speed for the exercises will be the maximum possible in each repetition.^[89,91]^ With regard to aerobic training, continuous aerobic training will be performed starting in the familiarization phase with a volume of 15 min (at 65% of the Heart-Rate Reserve [HRR]) until reaching a volume of 25 min at 85% of the HRR in phase 3. Exercise intensity will be controlled with heart rate monitors (Polar V800 pulsometer) and the subjective Rating of Perceived Exertion (0–10 Borg) in the case of aerobic training, as well as the OMNI-Resistance Exercise Scale for strength training.^[92]^Table 3 represents the training program schematically.

**Table 3:**
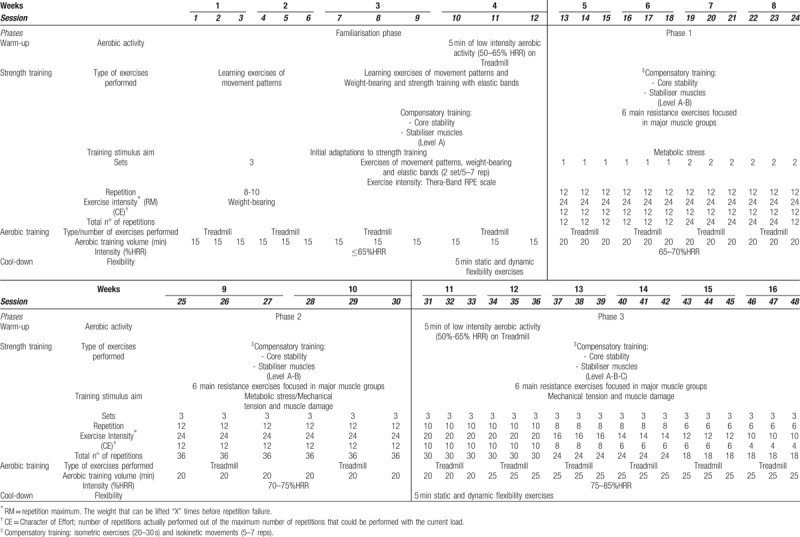
Training periodization.

##### 2.7.1.1 Strategies to register and maximize adherence to the training program

Adherence to the exercise program will be measured throughout the intervention period on a record sheet designed *ad hoc* and completed daily by the personal trainer in each session. In addition to the attendance percentage ([no. of sessions attended/no. of sessions planned] × 100), other variables such as punctuality, physical activity outside of the program, the number and type of adverse events, and the compliance attitude during the session will also be recorded. The level of perceived effort, mood and sense of acute exhaustion induced by exercise will be recorded to try to anticipate possible symptoms (if any) of fatigue or dissatisfaction and to make pertinent adaptations (if needed). Since program adherence is essential to study the clinical effectiveness of the exercise program, strategies will be implemented to maximize it, such as sending motivational messages (each week) and videos (each month) via Whatsapp throughout the program intervention.^[84]^

#### 2.7.2 Control/usual care group (CG)

All participants (both in the CG and EG) will receive the usual hygiene-dietary and healthy-cardio lifestyle recommendations in line with international guidelines.^[27]^ Patient's lifestyle following bariatric surgery is fundamental to its success, so all participants will receive both at the time of medical discharge and during post-operative visits (at 1, 3, 6, 9, and 12 months; see Fig. 2) advice based on the latest evidence-based clinical recommendations, jointly established by the American Association of Clinical Endocrinology, the Obesity Society, and the American Society of Bariatric and Metabolic Surgery.^[27]^

### 2.8 Participant care during the follow-up (from T2 to T3)

From the end of the intervention (week 16; T2) until the 12-month follow-up assessment (T3; approximately week 52 after surgery), participants in both groups will receive exactly the same treatment, that is, their usual follow-up (see Fig. 2) following bariatric surgery. Between T2 and T3, the EG will not receive any additional information on physical activity, exercise, or any aspect as distinct from the CG. In this way, we will be able to study the medium-term effect (12 months) of the 16-week exercise program.

### 2.9 Patient safety

For the entire study period, any possible adverse effects attributable to the intervention will be reported. An adverse effect in the EG is defined as any skeletal muscle injury that occurs during (or as a result of) the training performed.

### 2.10 Criteria for interrupting study participation

The number of participants interrupting participation, together with the reasons, will be registered and appropriately reported.

#### 2.10.1 Abandonment by the participant

Any participant may leave the study at any time without having to give an explanation and without any consequence to the medical care or treatments received by any agent related to the study.

#### 2.10.2 Interrupting participation by the researcher

The research team may discontinue any patient's participation (a withdrawn participant) at any time, provided that their participation poses a risk to their safety or a violation of the study protocol. This can occur under the following circumstances:

- Severe skeletal muscle injury that alters one's normal lifestyle (as a consequence, or not, of participating in the study).
- Pregnancy during the intervention period. Alterations in the hormonal profile that occurs during pregnancy would irredeemably affect the study results.
- A change of residence, making all postoperative assessments impossible.
- Death.

### 2.11 Statistical analysis

Despite randomization, the between-group baseline comparability will be checked. Normal distribution of continuous quantitative variables will be graphically inspected using histograms, and—when needed—normality tests will also be used. The between-group differences in the change (post–pre) in the primary and secondary effects (the main and secondary objectives 1, 2, and 3) will be analysed using a general linear model (in the case of significant deviation from normality, we will proceed with non-parametric techniques, such as quantile regression). The effect size (95% confidence interval) and the level of statistical significance will be displayed. In order to demonstrate the clinical efficacy of the supervised exercise program on ovarian function after bariatric surgery, the main analysis will be a per-protocol analysis (i.e., it will only include participants who attend at least 80% of the exercise sessions). Sensitivity analyses will be carried out using the intention-to-treat principle to check the robustness of the results. Missing data will be replaced using multiple account imputation. Despite the randomization, we will attempt to minimize potential confounding by adjusting for baseline values and other potential confounders. The association between changes in ovarian function with changes in the mechanisms involved (secondary objectives 4, 5, and 6) will be analysed using Pearson or Spearman correlations and/or regression analysis. The analyses will be conducted with Stata v.13.1 or superior (StataCorp LP, College Station, TX). Statistical significance will be set at *P* < .05.

### 2.12 Registration and adherence to the SPIRIT standards

The study has been prospectively registered at the ISRCTN registry (ISRCTN27697878) on October 4, 2019, before the enrolment of participants begun (i.e., on October 15, 2019). This study adheres to the SPIRIT guidelines for randomized trials protocols^[93]^ and the results will be reported following the CONSORT standards (http://www.consort-statement.org/).

## 3 Discussion

To the best of our knowledge, the EMOVAR study will assess, for the first time, the effects of a fully supervised concurrent exercise program initiated immediately after bariatric surgery, on ovarian function in severe/morbid obese women undergoing bariatric surgery. Previous studies demonstrate a U-shaped association of exercise with ovulation, with sedentary and over-trained women presenting higher incidences of anovulation.^[52]^ This relationship seems to be accentuated as BMI increases,^[52]^ underlining the importance of addressing the optimal dose of exercise. However, exercise prescription in morbid obese individuals is far from being well understood and little is known about the optimal dose of exercise in the morbid obese following bariatric surgery. We propose an evidence-based exercise intervention^[84]^ and describe it so that any agent involved in the management of morbid obese or bariatric patients can implement it in clinical practice, provided appropriate equipment and exercise professionals are available. This has been traditionally lacking in exercise-based clinical trials^[94]^ and opens a window of opportunity to translate exercise-based research into clinical practice. It is of course apparent that previous exercise-based literature in bariatric surgery individuals is clearly insufficient, and we recognize that some criteria are derived from studies carried out in non-morbid obese (nor bariatric surgery) individuals. For instance, the order of factors in the concurrent training (i.e., strength before aerobic training) was determined according to studies in obese (but not bariatric surgery) population, in which this order was the most beneficial for improving body composition, physical fitness and other health biomarkers.^[41]^ We also carried out a 2-week pilot exercise program to test the feasibility of some exercises in people with different characteristics who had undergone bariatric surgery in the prior weeks, which allowed our team adapting the initially prescribed program to the current structure.^[84]^ Therefore, the results of the EMOVAR clinical trial will have important clinical implications, not only for understanding the potential effects of exercise on ovarian function following bariatric surgery, but also for setting the bases of exercise prescription in this population.

Should the prescribed exercise program initiated immediately after bariatric surgery not have the expected beneficial effect on ovarian function, inflammation and arterial stiffness compared to usual care, the results of this clinical trial would still be of wide interest for the scientific community. These results could be explained by the great potential of bariatric surgery for improving weight loss during the first year following surgery. Should such unexpected results occur, this study would still contribute to the generation of new research ideas and hypotheses, such as modifying exercise program characteristics or combine it with other interventions such as nutritional or psychological treatments (or both), or extending the follow-up.

The trial results will be disseminated through international journals and other sources, without restrictions. Participants will receive personal information of their own results as well as a summary of the overall trial results.

### 3.1 Study limitations/risks and contingency plan

Potential risk 1: EG patients do not attend the intervention.

Contingency plan: Different strategies will be implemented to maximize adherence to the exercise program. We will have a Contracted Personal Trainer with a Bachelor's or graduate degree in Physical Activity and Sports Sciences and a Master's in Personal Training, who has experience in training people with obesity. Each week, the EG participants will receive motivational messages via WhatsApp. In addition, at the start of each month, patients will receive a motivational video from the coaches and medical team through the same channels. The emotional state (The Feeling Scale) will be monitored and any adverse events that occur will be recorded during training. This will allow us to understand how they feel and offer alternatives.

Potential risk 2: Recruitment and/or retention rate are lower than planned.

Contingency plan: With the medical team involved, a large proportion of childbearing age women undergoing bariatric surgery in the province of Almería will be invited to participate in the study. If the rate of recruitment is not as expected, we would try to arrange new exercise training facilities (other than the University of Almería) in areas of the province where a greater number of participants could be included.

## Author contributions

**Conceptualization:** Alberto Soriano-Maldonado, Manuel Ferrer-Márquez, Enrique G. Artero, Ana M. Fernández-Alonso.

**Formal analysis:** Alberto Soriano-Maldonado, Enrique G. Artero, Ana M. Fernández-Alonso.

**Funding acquisition:** Alberto Soriano-Maldonado, Ana M. Fernández-Alonso.

**Investigation:** Alberto Soriano-Maldonado, Sonia Martínez-Forte, Manuel Ferrer-Márquez, Elena Martínez-Rosales, Alba Hernández-Martínez, Alejandro Carretero-Ruiz, Emilio Villa-González, Yaira Barranco-Ruiz, Manuel A. Rodríguez-Pérez, María José Torrente-Sánchez, Lorena Carmona-Rodríguez, Pablo Soriano-Maldonado, José A. Vargas-Hitos, Antonio J. Casimiro-Andújar, Enrique G. Artero, Ana M. Fernández-Alonso

**Methodology:** Alberto Soriano-Maldonado, Manuel Ferrer-Márquez, Enrique G. Artero, Ana M. Fernández-Alonso.

**Project administration:** Alberto Soriano-Maldonado, Ana M. Fernández-Alonso.

**Resources:** Alberto Soriano-Maldonado, Sonia Martínez-Forte, Manuel Ferrer-Márquez, Lorena Carmona-Rodríguez, Pablo Soriano-Maldonado, Enrique G. Artero, Ana M. Fernández-Alonso.

**Software:** Alberto Soriano-Maldonado,

**Supervision:** Alberto Soriano-Maldonado, Ana M. Fernández-Alonso.

**Validation:** Alberto Soriano-Maldonado, Manuel Ferrer-Márquez, Enrique G. Artero, Ana M. Fernández-Alonso.

**Visualization:** Sonia Martínez-Forte, Elena Martínez-Rosales, Alba Hernández-Martínez, Alejandro Carretero-Ruiz.

**Writing – original draft:** Alberto Soriano-Maldonado, Ana M. Fernández-Alonso.

**Writing – review and editing:** Alberto Soriano-Maldonado, Sonia Martínez-Forte, Manuel Ferrer-Márquez, Elena Martínez-Rosales, Alba Hernández-Martínez, Alejandro Carretero-Ruiz, Emilio Villa-González, Yaira Barranco-Ruiz, Manuel A. Rodríguez-Pérez, María José Torrente-Sánchez, Lorena Carmona-Rodríguez, Pablo Soriano-Maldonado, José A. Vargas-Hitos, Antonio J. Casimiro-Andújar, Enrique G. Artero, Ana M. Fernández-Alonso

Alberto Soriano-Maldonado orcid: 0000-0002-4626-420X.
